# Metagenomic Comparison of Two *Thiomicrospira* Lineages Inhabiting Contrasting Deep-Sea Hydrothermal Environments

**DOI:** 10.1371/journal.pone.0013530

**Published:** 2010-10-21

**Authors:** William J. Brazelton, John A. Baross

**Affiliations:** School of Oceanography and Center for Astrobiology and Early Evolution, University of Washington, Seattle, Washington, United States of America; Universidad Miguel Hernandez, Spain

## Abstract

**Background:**

The most widespread bacteria in oxic zones of carbonate chimneys at the serpentinite-hosted Lost City hydrothermal field, Mid-Atlantic Ridge, belong to the *Thiomicrospira* group of sulfur-oxidizing chemolithoautotrophs. It is unclear why *Thiomicrospira*-like organisms thrive in these chimneys considering that Lost City hydrothermal fluids are notably lacking in hydrogen sulfide and carbon dioxide.

**Methodology/Principal Findings:**

Here we describe metagenomic sequences obtained from a Lost City carbonate chimney that are highly similar to the genome of *Thiomicrospira crunogena* XCL-2, an isolate from a basalt-hosted hydrothermal vent in the Pacific Ocean. Even though *T. crunogena* and Lost City *Thiomicrospira* inhabit different types of hydrothermal systems in different oceans, their genomic contents are highly similar. For example, sequences encoding the sulfur oxidation and carbon fixation pathways (including a carbon concentration mechanism) of *T. crunogena* are also present in the Lost City metagenome. Comparative genomic analyses also revealed substantial genomic changes that must have occurred since the divergence of the two lineages, including large genomic rearrangements, gene fusion events, a prophage insertion, and transposase activity.

**Conclusions/Significance:**

Our results show significant genomic similarity between *Thiomicrospira* organisms inhabiting different kinds of hydrothermal systems in different oceans, suggesting that these organisms are widespread and highly adaptable. These data also indicate genomic processes potentially associated with the adaptation of these lineages into strikingly different habitats.

## Introduction

Microbial oxidation of sulfur is the basis of most ecosystems at seafloor hydrothermal environments. In basalt-hosted hydrothermal vents, hydrogen sulfide (H_2_S) is the most abundant electron donor driving primary production [1]. Concentrations of H_2_S are ∼8 mmol/kg in typical basalt-hosted systems and can reach >40 mmol/kg at some sites [2]. Much of the animal biomass in these systems is directly supported by symbiotic H_2_S-oxidizing bacteria. At the serpentinite-hosted Lost City hydrothermal field in the Atlantic Ocean, H_2_S concentrations are lower [*2*], ranging between 0.05–2.8 mmol/kg in end-member hydrothermal fluids [*3*]. Nevertheless, mussels collected from Lost City chimneys harbor endosymbionts with close phylogenetic relationships to H_2_S-oxidizing as well as methane-oxidizing bacteria [*4*]. Megafaunal biomass is much lower at Lost City compared to most basalt-hosted systems, however, and the relative lack of H_2_S is the most likely cause [*2*]. Because fluid chemistry at Lost City is dominated by subsurface serpentinization reactions, hydrogen (H_2_, 1-15 mmol/kg) and methane (CH_4_, 1-2 mmol/kg) are much more abundant in chimney fluids than H_2_S [*2*]. Accordingly, archaea related to methanogens and methanotrophs comprise >80% of all detectable cells in biofilms associated with the hot, anoxic interiors of actively venting chimneys [5].

Bacteria are more abundant in biofilms attached to the outer walls of Lost City chimneys where hydrothermal fluids mix with cold, oxygenated seawater [5]. The most commonly detected bacteria in Lost City chimneys and fluids belong to the *Thiomicrospira* genus of *Gammaproteobacteria* [6], [7]. *Thiomicrospira* species frequently inhabit zones of hydrothermal chimneys and sediments where H_2_S and oxygen are both present [8], [9]. The basalt-hosted hydrothermal systems in which *Thiomicrospira* species are typically found are characterized by acidic fluids that contain abundant H_2_S and carbon dioxide (CO_2_). In contrast, the fluids exiting from Lost City chimneys are alkaline (pH 9–11), contain only moderate amounts of H_2_S, and are nearly devoid of CO_2_ [2], [10]. Nevertheless, the most prevalent bacterial 16S rRNA sequences in these fluids are affiliated with genus *Thiomicrospira* [6]. No Lost City *Thiomicrospira* have yet been cultivated, and it is unknown how they have adapted to these extreme environmental conditions. The relatively low H_2_S concentrations in Lost City fluids may not present serious difficulties for *Thiomicrospira* organisms because cultivated strains are known to grow optimally at H_2_S concentrations <1 mM [11]. The *Thiomicrospira* representatives at Lost City must harbor adaptations to the extremely low CO_2_ concentrations and high pH of Lost City fluids, however.

Here we compare metagenomic data from a Lost City carbonate chimney containing a large number of *Thiomicrospira*-related sequences to the only completed genome sequence of a *Thiomicrospira* organism: *Thiomicrospira crunogena* XCL-2 [*12*], which was isolated from diffuse fluids at the Galapagos Rift [*13*], a basalt-hosted hydrothermal system in the Pacific Ocean. H_2_S concentrations (∼0.2 mmol/kg) in fluids venting from surface sediments at the Galapagos Rift are diluted due to extensive mixing with seawater, and H_2_S levels in subsurface sediments are estimated to be much greater [*14*]. Additional strains with nearly identical 16S rRNA sequences to that of *T. crunogena* XCL-2 [15] have been isolated from basalt-hosted systems with H_2_S concentrations around 3–7 mmol/kg [16]. In contrast, H_2_S in Lost City end-member fluids never exceeds 2.8 mmol/kg and is much lower within carbonate chimneys where end-member fluids mix with ambient seawater [3]. Lost City fluids also have higher pH and lower CO_2_ concentrations than these basalt-hosted systems [16], [2]. Thus the metagenomic dataset described here provides a revealing snapshot of genomic changes associated with the divergence of two lineages into geochemically distinct habitats.

## Methods

### DNA extraction

The data described here are a subset of the dataset first reported in [17]. The carbonate chimney sample (H03_072705_R0424) was collected from the central ‘Poseidon’ edifice of the Lost City Hydrothermal Field (depth, 735 m; latitude, 30.12; longitude, −42.12) on 27 July 2005 by the DSV *Hercules* during the 2005 Lost City Expedition aboard the R/V *Ronald H. Brown*. Chimney material was frozen at −80°C immediately after collection and remained frozen until onshore analysis. DNA was extracted according to a protocol modified from previous reports [6], [18] and summarized here. After crushing a frozen carbonate sample with a sterile mortar and pestle, approximately 0.25–0.5 g of chimney material were placed in a 2 mL microcentrifuge tube containing 250 µL of 2x buffer AE (200 mM Tris, 50 mM EDTA, 300 mM EGTA, 200 mM NaCl, pH 8) and 2 µg of poly-dIdC (Sigma-Aldrich) and incubated at 4°C overnight to allow chelation of salts and binding of DNA to poly-dIdC. Between 36–72 replicate tubes were processed in parallel, and a total of ∼1 kg of carbonate minerals were processed. This protocol involved no size fractionation between sample collection and DNA extraction. Proteinase K (final concentration 1.2 mg/mL) and 10 µL of 20% SDS were added to each tube before incubation at 37°C for at most 30 min. A further 150 µL of 20% SDS and 500 µL of phenol∶chloroform∶isoamyl alcohol (25∶24∶1 ratio by volume) were added to each tube before centrifugation at 12,000 g for 10 min. Supernatants were transferred to clean tubes for a second phenol:choloroform:isoamyl alcohol extraction. After centrifugation, supernatants were pooled into SnakeSkin dialysis tubing (Pierce Protein Research Products; Rockford, IL) and dialyzed against 20 mM EGTA overnight at 4°C. This large scale dialysis step proved to be very efficient in removing minerals and organic inhibitors. After dialysis, DNA was precipitated by adding 0.1 vol 3 M sodium acetate and 1 vol isopropanol and stored at −20°C for 2–4 hours. Pellets were collected by centrifugation at 16,000 g for 20 min at 8°C, washed once in 70% ethanol, dried in a vacuum centrifuge, and resuspended in TE (10 mM Tris, 1 mM EDTA, pH 8). Typical yield was ∼35 ng of DNA per g of carbonate chimney material.

### Metagenomic sequencing and annotation

Library construction and Sanger end-sequencing of pUC18 inserts was conducted according to the standard protocols at the DOE Joint Genome Institute in 2007. Two libraries were constructed from two subsamples of the same carbonate chimney sample. Reads from both libraries were combined for assembly and for analyses described here. Mean read length for the 46 361 reads from both libraries was 755 bp, and the mean length of all 6324 contigs was 1583 bp. All sequencing reads are deposited under GenBank accession numbers ACQI01006325–ACQI01026573, and assembled contigs are deposited under accession numbers ACQI01000001–ACQI01006324. End-paired sequences are those with identical names and different suffix: *eg.* FNHG1000.b1 and FNHG1000.g1; FOSS3464.x1 and FOSS3464.y1. Open reading frames were assigned with Glimmer [19] and compared with *T. crunogena* genes by blastp [20]. Potential homologs for all *T. crunogena* proteins were identified by searching against a database of all Lost City metagenomic contigs or all unassembled sequencing reads with tblastn [20]. Visualization of BLAST results was facilitated by use of Artemis [21]. An Artemis file containing annotated open reading frames for Lost City metagenomic contigs is available at http://www.staff.washington.edu/braz. Files enabling identification of paired end sequences and membership of sequences in contigs are also available at the above website.

## Results and Discussion

### Comparison of genomic content

**Figure 1:**
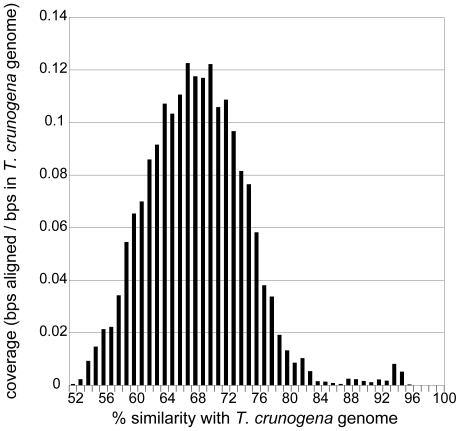
Most metagenomic reads from a Lost City carbonate chimney have moderate sequence similarity with *Thiomicrospira crunogena* XCL-2. Shotgun reads with >500 bp BLASTN alignments with the *T. crunogena* genome are binned according to the nucleotide sequence similarity (x-axis) of the BLASTN alignments. The y-axis represents the *T. crunogena* coverage for the reads in each bin where coverage corresponds to the ratio of the total number of aligned base pairs in each bin to the total number of base pairs in the *T. crunogena* genome, as in Figure 2 of [31].

As previously reported [17], we obtained 35 Mb of metagenomic sequence from 46 361 shotgun reads of two pUC18 libraries constructed by the DOE Joint Genome Institute with DNA extracted from ∼1 kg of a single Lost City carbonate chimney sample. A large proportion of the total shotgun reads (14.6%) had BLASTN alignments >500 bp with the *Thiomicrospira crunogena* XCL-2 genome [12]. Most of these reads exhibited 67–71% nucleotide similarity with *T. crunogena*, and very few reads contained sequences with >84% similarity (Figure 1). These data indicate that the Lost City metagenomic dataset is dominated by a population that is genotypically uniform and contains moderate sequence similarity with *T. crunogena*.

**Figure 2:**
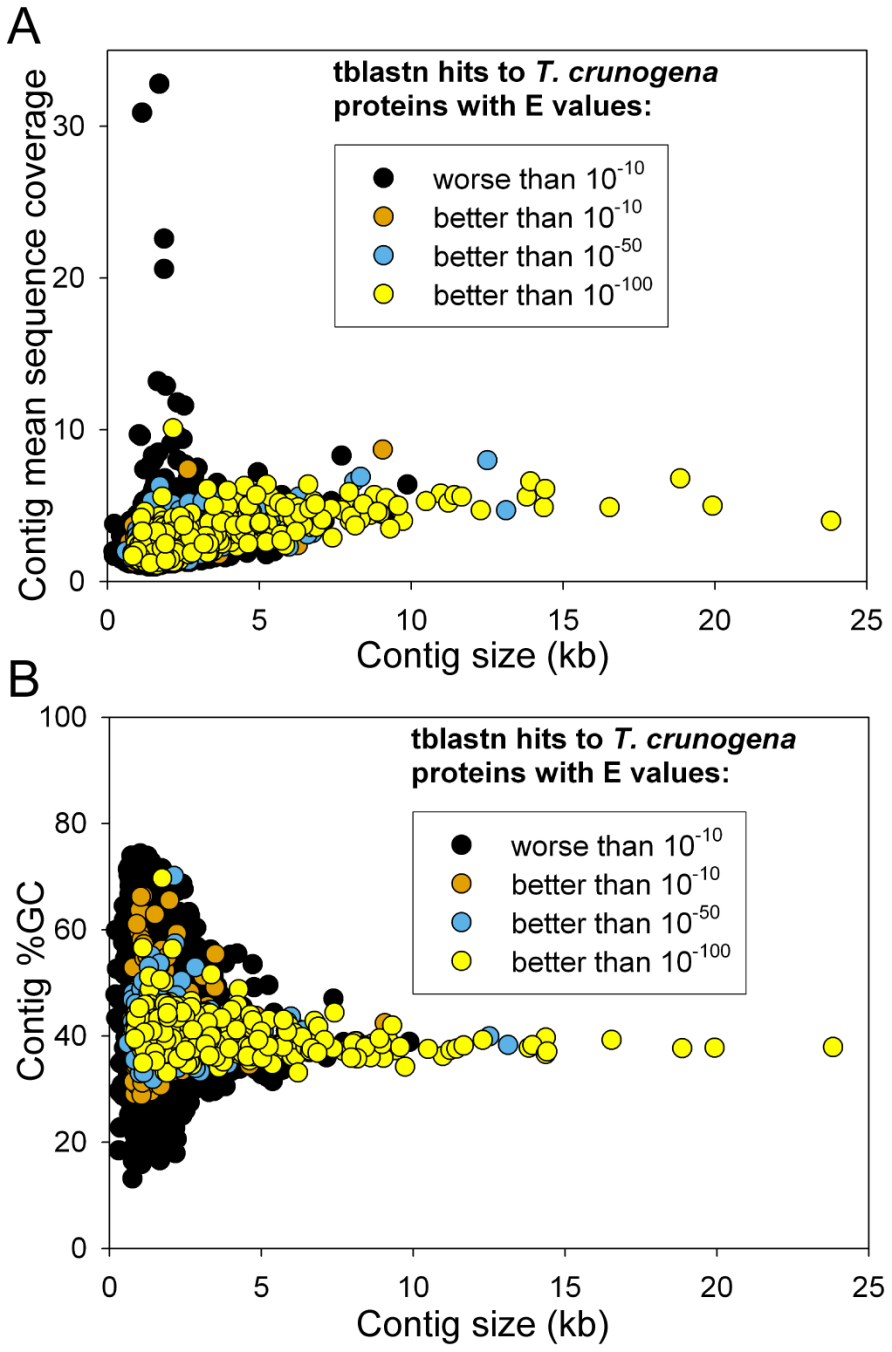
The Lost City *Thiomicrospira* population is represented by the largest metagenomic contigs. (A) All contigs larger than 10 kb have tblastn hits with at least one *T. crunogena* protein with an E value better than 10^−50^. These large contigs are present at 5–8x sequencing coverage. (B) Large contigs with *T. crunogena*-related sequences are ∼38%GC; the *T. crunogena* genome is 43%GC.

Approximately half of the shotgun reads assembled into 6324 contigs, including 49 contigs >7 kb in length. Almost all of the large contigs contained open reading frames with significant sequence similarity to a *Thiomicrospira crunogena* XCL-2 protein, indicating that *Thiomicrospira*-related sequences comprise a high proportion of the metagenomic assembly (Figure 2). The similar sequencing coverage of the largest contigs is consistent with a single population dominating the dataset (Figure 2A). These large contigs are ∼38%GC (Figure 2B), and the *T. crunogena* genome is 43%GC.

**Figure 3:**
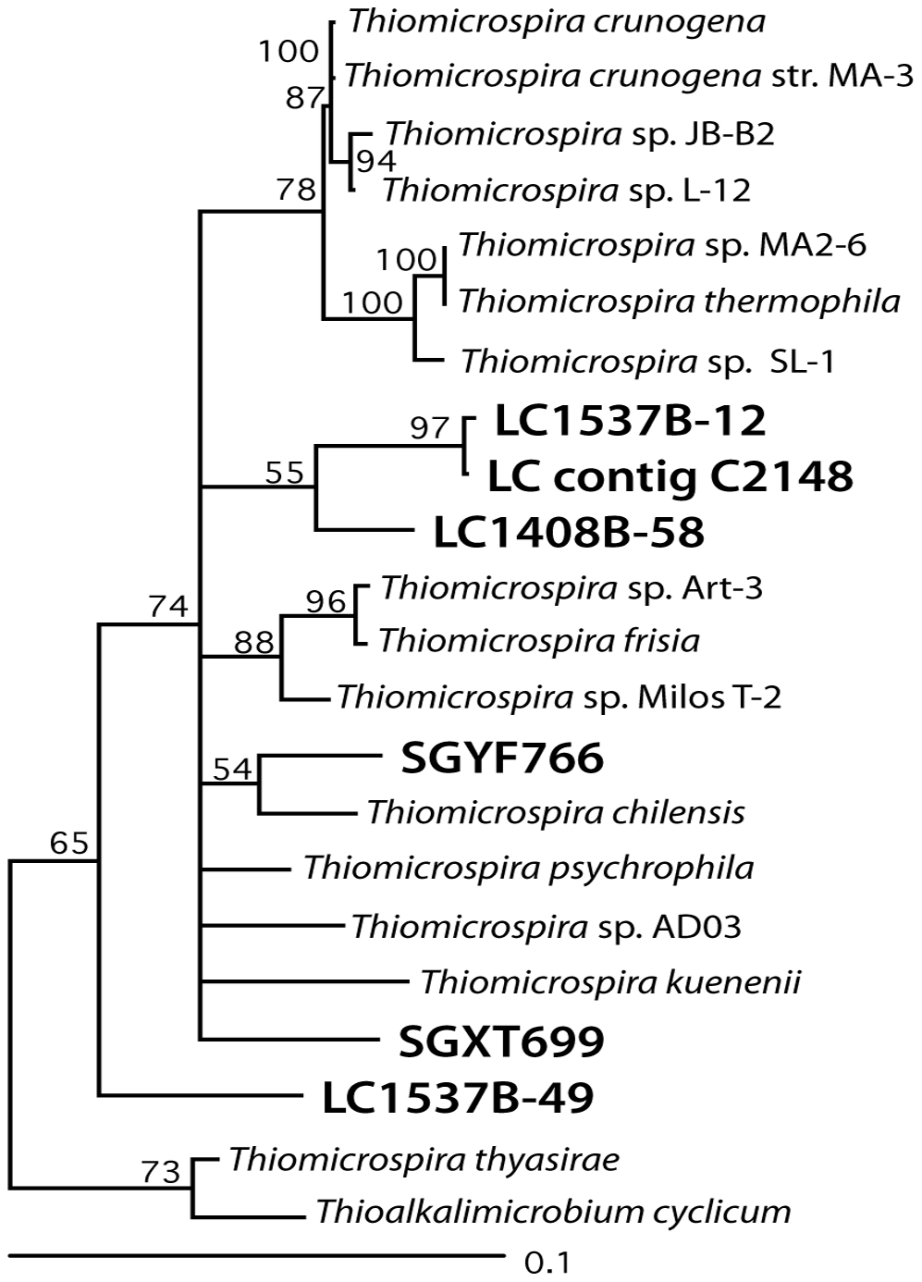
Phylogenetic tree of 16S rRNA sequences from *Thiomicrospira* cultivated isolates and clones collected from Lost City chimneys. Sequences with 100% identity to the V6 hypervariable region of clone LC1537B-12 and contig C2148 were found to be most abundant in a carbonate chimney aged 34 yrs venting fluids with a maximum temperature of 88°C [6]. Contig C2148 is 9 kb in length, has 42%GC, and has 8.7x coverage. The tree was constructed with Tree-Puzzle 5.2 [32] from 1278 characters aligned with MUSCLE [33]. Quartet puzzling support values are shown; nodes with less than 50% support are collapsed. Genbank accession numbers for each sequence from top to bottom: AF064545, AF069959, AF013972, AF064544, L40811, AB166731, AF013971, DQ270608, ACQI01002148, DQ270607, AF013973, AF013974, AJ237758, FJ792484, AF013975, AJ404732, AY575776, AF013978, FJ792098, DQ270609, AF016046, AF329082.

Our previous work has shown that multiple *Thiomicrospira* taxonomic units are present in Lost City chimneys, but the local conditions at each chimney determine which sequences are the most abundant [6]. The 16S rRNA sequence which dominates the youngest, warmest chimneys matches that in Lost City metagenomic contig C2148 (Figure 3). Nearly half of all bacterial 16S rRNA clones in a library constructed from the same DNA preparation used for metagenomic sequencing showed high similarity to *T. crunogena* (data reported in [17]), and half of these clones were nearly identical to the sequence in contig C2148. Therefore, the largest metagenomic contigs in this study are likely to represent a *Thiomicrospira* population that is abundant in the youngest, warmest Lost City carbonate chimneys. Although some Lost City chimneys vent fluids containing up to 2.8 mmol/kg H_2_S, the sample used in this metagenomic study was collected from a chimney venting <0.3 mmol/kg H_2_S (D. Butterfield, manuscript in prep.).

The large contigs contained surprisingly few archaeal sequences considering the dominance of archaea in actively venting Lost City chimneys [5]. Bacteria are known to be more abundant in the exterior, oxygenated zones of the chimneys [5], so the relative lack of archaeal sequences can be attributed to the sample containing little material from interior, anoxic zones of the chimney. These micro-scale redox zones are difficult to identify by bulk mineralogy due to pervasive mixing of ambient seawater through the highly porous carbonate chimneys [22].

**Figure 4:**
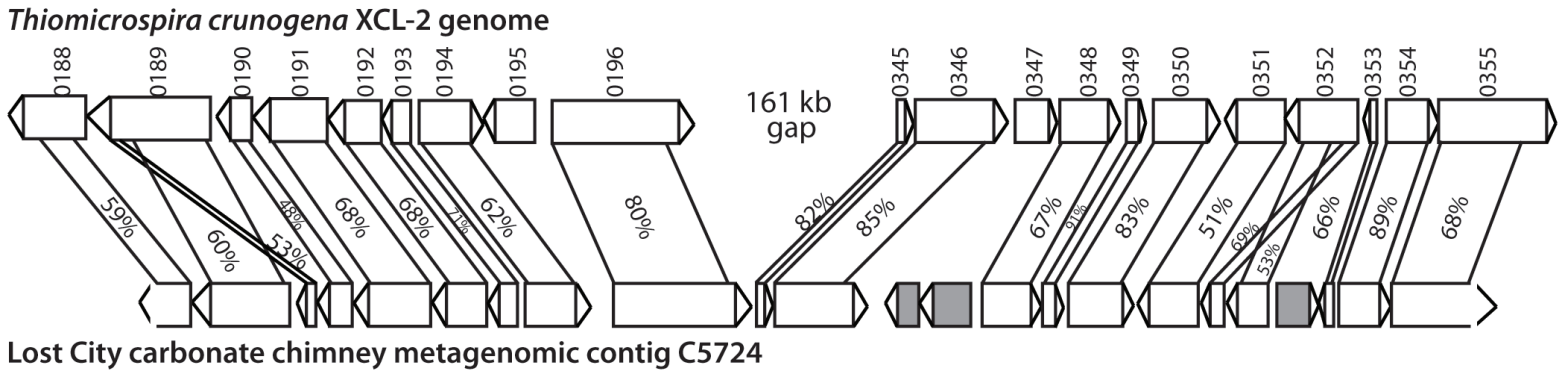
Conservation of gene order (synteny) between open reading frames (ORFs) in the *T. crunogena* genome (top) and the largest Lost City chimney metagenomic contig (bottom). The Lost City contig has putative homologs in two distinct regions of the *T. crunogena* genome separated by 161 kb. Amino acid identities and alignment lengths (as calculated by blastp) are shown. *T. crunogena* ORFs Tcr_0189 and Tcr_0352 each appear to be fusions of two Lost City ORFs. The three Lost City ORFs shaded gray are more similar to proteins from other bacteria including *Methylophaga* and *Marinobacter* and are in reverse orientation with respect to nearby ORFs. Accession number for contig C5724 is ACQI01005724.

Many genes in the *T. crunogena* XCL-2 genome [12] have putative orthologs in the Lost City metagenome; a complete list is available in the Supplementary Information as Table S1. Of the 2200 *T. crunogena* protein-coding genes (obtained from the Joint Genome Institute IMG database), 652 had tblastn [20] hits with E values better than 10^−100^ to a Lost City contig or unassembled sequence (1217 hits better than 10^−50^; 1842 hits better than 10^−10^). Of the 358 *T. crunogena* proteins lacking hits with E values better than 10^−10^, 214 were annotated as hypothetical proteins. Gene order is also highly conserved between the Lost City contigs and the *T. crunogena* genome, as exemplified by the largest contig (Figure 4). It is clear that large genomic rearrangements have occurred since the divergence of the two lineages, however, as the Lost City contig is syntenic with two distinct *T. crunogena* regions separated by 161 kb (Figure 4). Interestingly, three of the open reading frames (ORFs) in this contig encode proteins that are more similar to other bacteria (*Methylophaga*, *Marinobacter*) that have been identified in Lost City chimneys and fluids [6] and are in reverse orientation with respect to surrounding ORFs. Assembly error is an unlikely explanation for this result because in each case 3–6 sequencing reads contained at least one of these three ORFs as well as a nearby ORF with high sequence similarity to *T. crunogena*. These observations are consistent with an origin of these ORFs by lateral gene transfer, but further sequencing and phylogenetic analyses are required to test this hypothesis.

**Figure 5:**
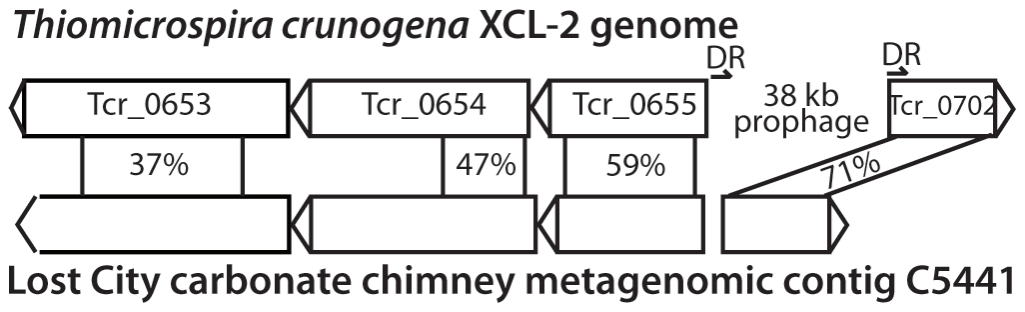
The prophage genome identified in *T. crunogena* is not present in the Lost City metagenome. ORFs on both sides of the prophage are present in Lost City metagenomic contig C5441 (accession # ACQI01005441), but a possible direct repeat (upstream region has 88% identities with positions 24–49 of Tcr_0702) is absent. Amino acid identities and alignment lengths (as calculated by blastp) are shown.

Another striking genomic difference between *T. crunogena* and Lost City metagenomic sequences is the presence of a prophage genome in *T. crunogena* but not in the corresponding Lost City contig (Figure 5). Genes flanking both ends of the *T. crunogena* prophage are present in the same Lost City contig, but no prophage sequences are present. Furthermore, a possible direct repeat caused by the insertion of the prophage genome into *T. crunogena* is also absent from the Lost City contig (Figure 5). If the prophage was previously present in the Lost City sequence and subsequently lost, the direct repeat is expected to have remained. Therefore, the absence of the prophage and direct repeat in the Lost City contig indicates that the prophage was inserted into *T. crunogena* after its divergence from the Lost City lineage.

### Comparison of sulfur utilization genes

**Figure 6:**
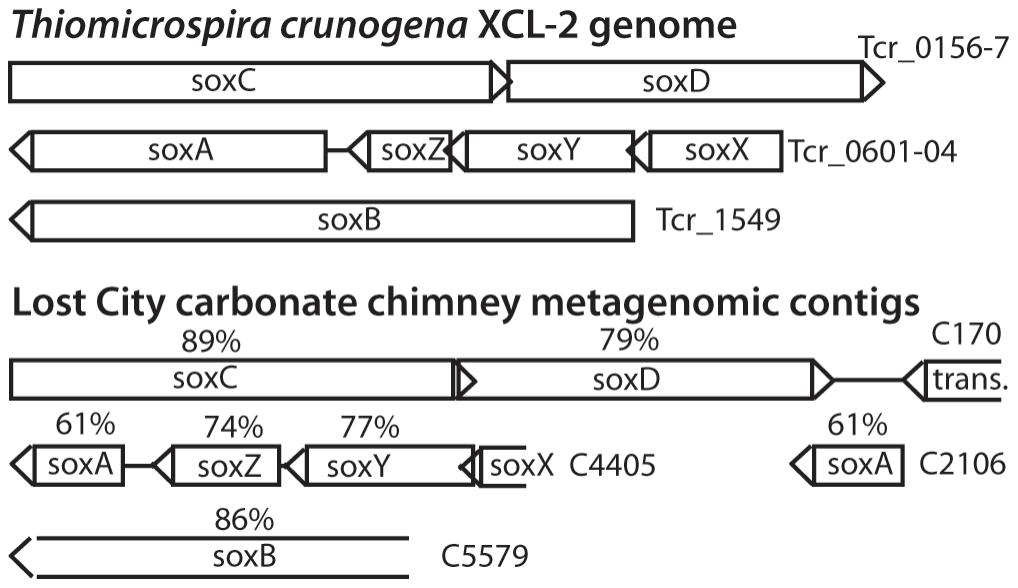
The Sox sulfur oxidation system of *T. crunogena* [**12**], which encodes all enzymes necessary for complete oxidation of H_2_S to sulfate, is also present in metagenomic sequences from Lost City. In most organisms utilizing the Sox pathway, all genes are organized into a single cluster, but in both *T. crunogena* and the Lost City metagenome, *sox*B and *sox*CD are not contiguous with *sox*XYZA. Amino acid identities between putative orthologs are shown; note that the Lost City metagenome contains an additional copy of *sox*A not associated with other *sox* genes, and both copies are only 61% identical to *T. crunogena* and only 90% identical to each other. The Lost City *sox*X and *sox*B sequences are incomplete. The presence of a transposase downstream of Lost City *sox*CD is of potential interest considering strong evidence indicating lateral transfer of sulfur oxidation genes among bacteria [23]. Accession numbers for contigs C170, C4405, C2106, and C5579 are ACQI01000170, ACQI01004405, ACQI01002106, and ACQI01005579, respectively.

The data presented thus far indicate that the Lost City *Thiomicrospira* population and *T. crunogena* represent two moderately closely related, but clearly distinct, lineages. We further examined the Lost City metagenomic data to examine whether these two lineages inhabiting contrasting hydrothermal systems contain similar sulfur utilization genes. *T. crunogena* utilizes the Sox pathway for complete oxidation of various sulfur compounds to sulfate [12]. We identified apparent homologs for all Sox genes required for sulfite-, thiosulfate-, elemental sulfur (S^0^), and H_2_S-dependent cytochrome c reduction in Lost City metagenomic contigs (Figure 6). Amino acid similarities between putative homologs range between 61% and 89%, and gene order appears to be conserved (Figure 6). Interestingly, *sox*B and *sox*CD are not contiguous with *sox*XYZA in both the *T. crunogena* genome and the Lost City metagenomic contigs. It is unclear whether this genomic arrangement has been maintained in both lineages by selection or if it is a result of recent evolutionary divergence.

In most organisms utilizing the Sox pathway, all genes are organized into a single cluster, and the few exceptions may have arisen as a result of lateral gene transfer [23]. Phylogenetic discrepancies between *T. crunogena* SoxCD (and the highly similar Lost City SoxCD) compared to the other Sox proteins are consistent with one or more lateral gene transfer events [23]. Considering this phylogenetic interpretation, the presence of a transposase near the Lost City *sox*CD sequences (Figure 6) is intriguing, and further work should investigate whether transfer of *sox*CD is mediated by transposases in Lost City chimneys. Scott *et al*. [12] have also suggested that the fragmented arrangement of Sox genes in *T. crunogena* could be maintained by the lack of selection pressure for regulation of a constitutively expressed pathway. Thus the Sox genes in the Lost City metagenome may be constitutively expressed, and at least some of them were likely acquired by an ancestor common to *T. crunogena* via lateral transfer.

Additional details of sulfur utilization by Lost City *Thiomicrospira* organisms can be inferred from the metagenomic data. One Lost City unassembled read (GenBank ACQI01023028) contains an open reading frame with 81% amino acid identities to a putative sulfide:quinone reductase (SQR) encoded by *T. crunogena* gene Tcr_1170. In *Rhodobacter capsulatus*, SQR is known to catalyze the reduction of H_2_S to S^0^, which accumulates as S^0^ granules outside the cells, and S^0^ is also deposited extracellularly by *T. crunogena* under certain conditions [24]. Alternatively, sulfur deposition in *T. crunogena* may be the result of ineffectual interactions among SoxCD, SoxYZ and SoxB due to their differing phylogenies [23]. Intracellular S^0^ granules have been observed in unidentified filamentous microorganisms collected from Lost City chimneys [25], but extracellular S^0^ granules have not yet been reported.

The *T. crunogena* genome is also notable for its lack of genes encoding sulfate assimilation enzymes, indicating that it depends entirely on reduced sulfur species. This also appears to be true for the Lost City *Thiomicrospira* representative, as none of the metagenomic contigs with *Thiomicrospira*-related sequences contain ATP sulfurylase, APS kinase, or PAPS reductase. Apparent homologs for the latter two sequences were identified in very short contigs, but the taxonomic affiliation of these contigs is not readily apparent.

### Comparison of carbon fixation genes

**Figure 7:**
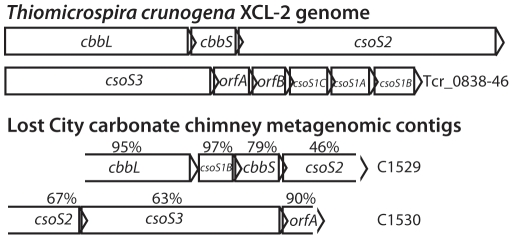
Most of the genes in the carboxysome operon of *Thiomicrospira crunogena* are present in Lost City metagenomic sequences. Amino acid percent identities between putative homologs are shown. The Lost City sequences are incomplete; it is expected that further sequencing and assembly will yield a complete carboxysome operon. *cbb*L and *cbb*S encode the large and small subunits of RubisCO. *cso*S3 encodes carbonic anhydrase. All other genes are expected to encode structural proteins forming the carboxysome shell. Accession numbers for contigs C1529 and C1530 are ACQI01001529 and ACQI01001530.

Considering the very low CO_2_ concentrations in Lost City chimney fluids [2], [10], it is expected that Lost City *Thiomicrospira* organisms harbor adaptations for living in a low CO_2_ environment. Indeed, we identified metagenomic sequences encoding a partial carboxysome operon including genes for RubisCO, carboxysome shell proteins, and carbonic anhydrase (Figure 7). Carboxysomes are protein microcompartments in which CO_2_ is concentrated to optimize carbon fixation by RubisCO. The carbonic anhydrase gene present in the carboxysome operon of *T. crunogena* is transcribed more frequently under low CO_2_ conditions, consistent with its role in carbon concentration [26]. Phylogenetic analysis confirmed that the Lost City carbonic anhydrase is most closely related to this *T. crunogena* gene (Tcr_0841; data not shown), suggesting that it may be associated with adaptation to the low CO_2_ levels at Lost City. Also present in the Lost City metagenomic dataset is a SulP-type sulfate transporter (88% identities to Tcr_1533), which shares some sequence similarity with proteins involved in bicarbonate transport into the cell [27], but it is unknown whether this protein is responsible for generating elevated concentrations of intracellular bicarbonate in *Thiomicrospira*.

In the genomes of all obligate autotrophs including *T. crunogena*, RubisCO genes are not located near other enzymes of the Calvin-Benson-Bassham cycle [12]. In the Lost City metagenomic dataset, none of the contigs containing putative RubisCO genes also contain other sequences likely involved in the Calvin-Benson-Bassham cycle. Also, an open reading frame with high similarity to phosphoribulokinase of *T. crunogena* (Tcr_0013) is present in a Lost City contig without any RubisCO genes. This fragmented arrangement of carbon fixation genes is consistent with the Lost City *Thiomicrospira* representative being an obligate autotroph.

The genome of *T. crunogena* encodes three different RubisCO enzymes, two form I RubisCOs and one form II Rubisco [12]. The form II RubisCO is only expressed under high CO_2_ concentrations [27]. The Lost City metagenomic data includes apparent homologs for the two form I RubisCOs (Figure 7 and Table S1), but none of the assembled contigs contain a form II RubisCO. (Two unassembled sequences encode form II RubisCOs, but their closest relatives do not include *T. crunogena*). Although no firm conclusions can be drawn from the absence of genes in an incomplete metagenomic dataset, it appears that the low CO_2_ concentrations in Lost City fluids has rendered the high-CO_2_ form II RubisCO unnecessary for Lost City *Thiomicrospira*.

### Comparison of transposase sequences

We have previously observed that the Lost City metagenome contains a surprisingly high abundance and diversity of sequences that encode transposases [17]. The transposase sequences were found in all of the smallest, highest coverage contigs and were rarely found in large contigs, suggesting an origin from viruses or extragenomic molecules. Another possibility is that these contigs represent genomic regions that are not amenable to assembly into larger contigs. Genomic regions containing transposase sequences can be expected to be highly variable, and such non-consensus sequences could not be assembled into large contigs with the sequencing effort of this study. Therefore, it is plausible that many of the transposase-containing contigs identified in our earlier report [17] represent variable regions of the Lost City *Thiomicrospira* pangenome, which differ among closely related strains within the Lost City *Thiomicrospira* phylotype.

**Figure 8:**
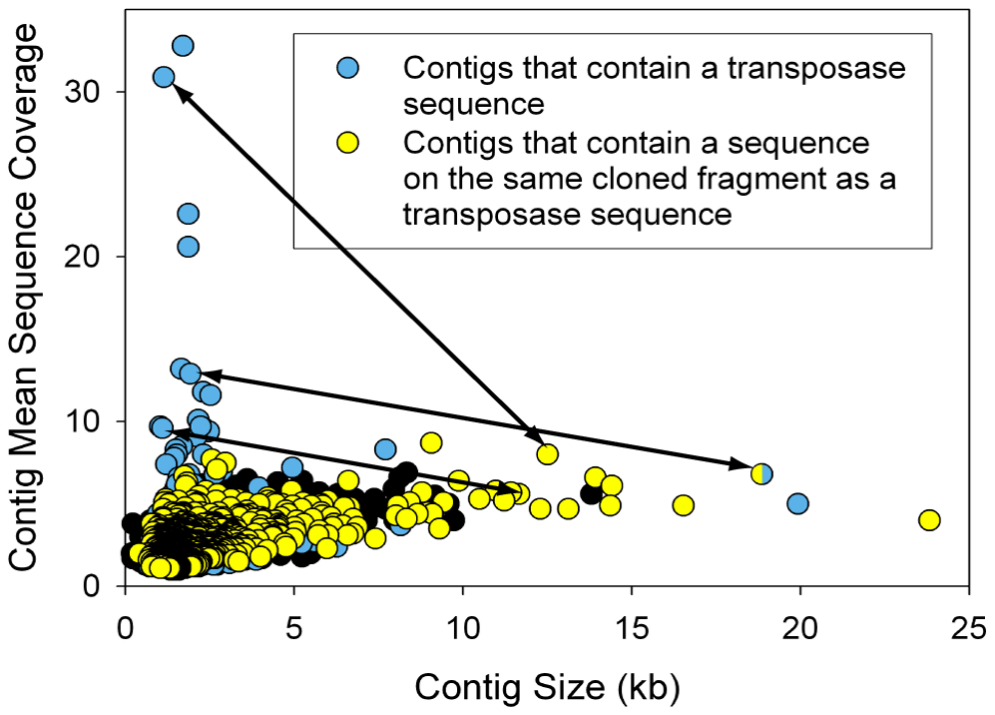
Contigs that encode transposases (blue points) are generally small and high-coverage. Yellow points represent contigs that do not encode transposases but contain at least one sequencing read from the end of a clone that contains a transposase on the opposite end. Arrows indicate examples of contigs linked by such paired end sequences. One of these examples involves a large contig that encodes a transposase and is also linked to a small transposase-containing contig (blue and yellow point). These links among contigs suggest that the Lost City *Thiomicrospira* pangenome contains a large number of transposase sequences.

To test this hypothesis, we examined whether the largest *Thiomicrospira*-like contigs are physically linked with unassembled transposase sequences; *i.e.* we searched for paired end sequences of the cloned metagenomic fragments where one member of the pair was assembled into a large contig and one member contained a transposase sequence. Figure 8 shows that 16 of the 17 contigs >10 kb contain at least one sequence paired with a transposase-containing sequence. Of the 1294 sequences that comprise these contigs, 49 (3.8%) were paired with a transposase sequence. If we conservatively estimate that a single transposase sequence is associated with each of the 16 contigs (244 kb), then a completed genomic assembly should contain one transposase per 15 kb. In comparison, *T. crunogena* contains only 20 transposases: 0.8% of the genome, or one per 120 kb (from the annotation at http://img.jgi.doe.gov). Although these results must be considered preliminary until a finished genome sequence is obtained, they are suggestive that the Lost City *Thiomicrospira* pangenome has a high transposase content.

This analysis also sheds some light on the nature of the extremely high-coverage, transposase-containing contigs (the top-left cluster in Figure 8). The arrows in Figure 8 indicate that three of these contigs are paired with sequences on three of the large, *Thiomicrospira*-like contigs. Therefore, these transposases are probably part of the Lost City *Thiomicrospira* genome, and their extremely high coverage (*eg.* >30x for contig C5672) indicates that they must be present in multiple copies. These examples are rare, however. Most of the high-coverage, transposase-containing contigs were not physically associated with the large, *Thiomicrospira*-like contigs, and their origin remains unclear. Future genomic sequencing may prove that these contigs, too, represent genomic regions present as multiple copies, but the present data are also consistent with the previous suggestion that they are derived from viruses or small extragenomic molecules [17].

### Ecological and evolutionary implications

Among the 358 *T. crunogena* genes without close relatives in the Lost City metagenome, a few have apparent ecological implications. For example, the phosphonate operon (encodes the ability to utilize organic phosphate) in *T. crunogena* is not syntenic with any Lost City contigs, and only some of the genes in the phosphonate operon appear to have homologs in Lost City sequences, In contrast, Lost City contigs contain sequences encoding both the low-affinity and high-affinity (inorganic) phosphate uptake systems present in *T. crunogena.* Three putative sulfonate transporters in *T. crunogena* are also missing in the Lost City sequences. Therefore, it is possible that Lost City *Thiomicrospira* are dependent on inorganic forms of phosphorus and sulfur, although no firm conclusions can be drawn due to the incomplete nature of the metagenome.

Previous studies have noted the inability of *T. crunogena* to utlize hydrogen gas (H_2_) as a sole electron donor despite the presence of a Ni/Fe hydrogenase operon in its genome [12], [28]. Considering the abundance of H_2_ in Lost City fluids [2], the ability of Lost City *Thiomicrospira* to utilize H_2_ with this hydrogenase operon is of interest. One contig in our dataset (C4437) appears to contain a partial Ni/Fe hydrogenase operon, but these sequences share little similarity with those in *T. crunogena* and most likely derive from other lineages. Due to the incomplete nature of the metagenomic dataset, however, we cannot conclude with certainty whether Lost City *Thiomicrospira* harbor hydrogenases.

Considering their apparent inability to utilize the most abundant electron donor (H_2_) and their dependence on two substrates (H_2_S and CO_2_) that are notably lacking in Lost City fluids, it is remarkable that *Thiomicrospira* are the most widespread and abundant bacteria in Lost City carbonate chimneys [6], [7]. The surprising dominance of these organisms is most likely related to patterns of oxygen and CO_2_ availability in carbonate chimneys. Those organisms expected to utilize H_2_ at Lost City (*Methanosarcinales*- and *Desulfotomaculum*-related organisms) are anaerobic and more prevalent in the anoxic, interior zones of carbonate chimneys where the aerobic *Thiomicrospira* are unlikely to survive [5], [6]. Pervasive mixing of oxygenated seawater throughout the highly porous structure of the carbonate chimneys [22] could explain the relative lack of *Epsilonproteobacteria*, as these organisms are typically restricted to areas with low oxygen concentrations [29]. *Thiomicrospira*, in contrast, can thrive in fully oxic environments as long as reduced sulfur species are present [8]. Furthermore, *Thiomicrospira* may out-compete *Epsilonproteobacteria* as well as all other bacteria in Lost City fluids because they harbor a carbon-concentrating mechanism that allows them to remain autotrophic at very low CO_2_ concentrations (Figure 7). Isotopic analyses indicate that bacteria in Lost City carbonate chimneys are extremely carbon-limited [30], so a carbon-concentration mechanism could be highly advantageous.

### Conclusions

In summary, metagenomic analyses of a Lost City carbonate chimney reveal a dominant *Thiomicrospira* population with similar genomic content to a similar but distinct species, *Thiomicrospira crunogena* XCL-2. Despite inhabiting different types of hydrothermal systems in different oceans, both *Thiomicrospira* representatives share genes encoding functions that appear to be crucial for thriving in Lost City carbonate chimneys: the ability to aerobically oxidize reduced sulfur species and to concentrate CO_2_ intracellularly. This genomic similarity likely reflects a recent evolutionary divergence and that both lineages inhabit niches where H_2_S-containing hydrothermal fluids mix with oxygenated seawater.

Further sequencing and physiological experiments will be necessary to identify particular genomic differences associated with living in different environments, but the data presented here indicate that substantial genomic evolution has occurred since the divergence of these two lineages. For example, genetic recombination appears to have been a major factor, evidenced by the large break in synteny and two gene fusion events illustrated in Figure 4. Furthermore, the evolution of the *Thiomicrospira* lineage has been strongly influenced by lateral gene transfer, as there is strong evidence for lateral transfer events both before (SoxCD phylogeny; [23]) and after (prophage insertion; Figure 3) the divergence of the Lost City *Thiomicrospira* population and *T. crunogena*. The extremely high transposase content of Lost City *Thiomicrospira* genomic regions (Figure 8) is also strongly suggestive that lateral gene transfer has played an important role in its evolution. Future experiments should investigate whether transposase activity has promoted diversification of the dominant Lost City *Thiomicrospira* phylotype into multiple strains or ecotypes, each harboring unique genomic rearrangements caused by transposases.

## Supporting Information

Table S1 Each of 2200 T. crunogena protein-coding genes were compared with Lost City metagenomic contigs and unassembled sequencing reads (singlets) with tblastn [20]. The Lost City contig and singlet with the best tblastn scores are shown for each T. crunogena protein.
(1.04 MB XLS)
